# Relativistic Compression and Expansion of Experiential Time in the Left and Right Space

**DOI:** 10.1371/journal.pone.0001716

**Published:** 2008-03-05

**Authors:** Carmelo Mario Vicario, Patrizia Pecoraro, Patrizia Turriziani, Giacomo Koch, Carlo Caltagirone, Massimiliano Oliveri

**Affiliations:** 1 Dipartimento di Psicologia, Università di Palermo, Palermo, Italy; 2 Dipartimento di Psicologia, Università di Roma La Sapienza, Rome, Italy; 3 Fondazione “Santa Lucia” Istituti di Ricovero e Cura a Carattere Scientifico (IRCCS), Roma, Italy; University of Birmingham, United Kingdom

## Abstract

Time, space and numbers are closely linked in the physical world. However, the relativistic-like effects on time perception of spatial and magnitude factors remain poorly investigated. Here we wanted to investigate whether duration judgments of digit visual stimuli are biased depending on the side of space where the stimuli are presented and on the magnitude of the stimulus itself. Different groups of healthy subjects performed duration judgment tasks on various types of visual stimuli. In the first two experiments visual stimuli were constituted by digit pairs (1 and 9), presented in the centre of the screen or in the right and left space. In a third experiment visual stimuli were constituted by black circles. The duration of the reference stimulus was fixed at 300 ms. Subjects had to indicate the relative duration of the test stimulus compared with the reference one. The main results showed that, regardless of digit magnitude, duration of stimuli presented in the left hemispace is underestimated and that of stimuli presented in the right hemispace is overestimated. On the other hand, in midline position, duration judgments are affected by the numerical magnitude of the presented stimulus, with time underestimation of stimuli of low magnitude and time overestimation of stimuli of high magnitude. These results argue for the presence of strict interactions between space, time and magnitude representation on the human brain.

## Introduction

Time perception is fundamental to many aspects of our lives. Most scientists agree that the explicit study of time falls in the purview of physics, yet time is an integral part of virtually all psychological phenomena.

A century ago Einstein's insight revolutionised physics, postulating the relativity of time in the physical world and its strict correlation with space. A similar conceptual approach is likely needed to understand the timing of visual events in the human brain.

In fact, a wealth of human and animal research has supported common processing of temporal information with other magnitudes [1], [2], [3], [4], [5]. Classical models [1], [6] suggest that using multiple switches and accumulators an organism could quantify time and number simultaneously. More recently, these models have been revised to postulate that numerical and temporal integration may be carried out by a distributed neural circuit that includes cortical areas activated by both timing and counting tasks [7].

As to the relations linking time with spatial factors, previous evidence [8] has documented an experiential relativity of perceived time in humans according to the environmental scale. Indeed, much work has shown that for the brain time and space are not processed separately but can influence each other strongly [9], [10].

All these findings are unified in a theory stating that time, space and quantity are part of a generalized magnitude system in the primates' brains, where specific cortical areas process these elements of the environment [11].

However, in spite of this growing interest in the functional interactions between time and other magnitudes, there is relatively little in the literature on how spatial, numerical and temporal dimensions interact in the cognitive system and on the parallel or hierarchical nature of such interactions.

Here, we used different ordered materials (temporal and numerical) in the same task to test whether relativistic-like effects could compress and expand perceptual time according to two factors: the magnitude of the stimulus; the side of space where a visual stimulus is presented.

## Results

When stimulus digits were presented in midline position, time perception was biased according to the magnitude of the digit: the duration of test low digits was underestimated compared with that of reference high digits and vice-versa, as revealed by the significant difference of bisection points in the two conditions (unpaired t-test: t = 12.2; p = 0.0001).

**Figure 1:**
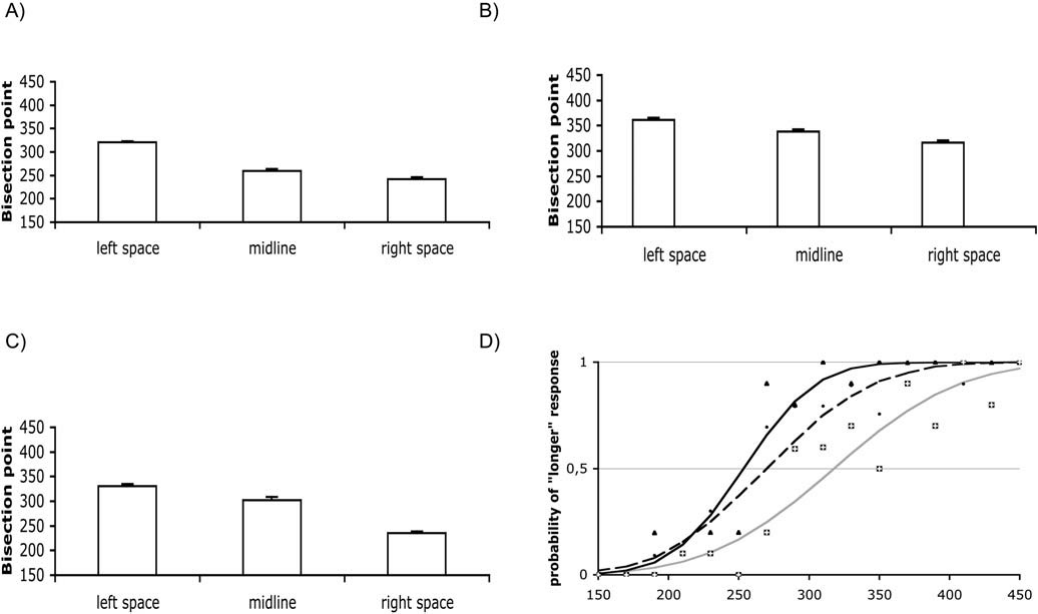
Duration distortions in the right and left space. (a) Experiment 1: average bisection points when digit 9 was the test stimulus. (b) Experiment 2: average bisection points when digit 1 was the test stimulus. (c) Experiment 3: average bisection points when circles were used as reference and test stimuli. (d) Representative data from one participant in Experiment 1: Black continuous line and black triangles: test stimulus in right space; black dashed line and black circles: test stimulus in central position; grey line and white squares: test stimulus in left space.

When stimulus digits were presented lateralised in the right and left visual hemifields, time perception was biased according to their spatial lateralisation: the “bisection” point was significantly longer in the left vs. right space, either when the digit 9 (paired t-test = 20.9; p = 0.0001) or the digit 1 (paired t-test = 8.8; p = 0.0001) was the test stimulus, as well as in the case of stimuli constituted by circles (paired t-test = 13.9; p = 0.0001) (Figure 1 A,B,C,D).

Duration perception of spatially lateralized stimuli was still influenced by numerical magnitude: in fact, bisection points were significantly shorter when the test stimulus was the digit 9 as compared with digit 1, either in the right space (unpaired t-test: t = 10.9; p = 0.0001) or in the left space (unpaired t-test: t = 7.7; p = 0.0001).

## Discussion

The current findings suggest that, as in the physical world, psychological time is relative and elastic. Time can be compressed and expanded by a number of environmental factors such as the position of a stimulus, on a left-to-right vectorial distribution, or its magnitude.

The relation between time and numbers seems to be influenced by numerical quantity: small numbers bias estimation towards short durations whereas large numbers bias estimation towards long durations in a behavioral standard time comparison task. These findings argue for a functional interaction between number magnitude processing and time estimation. The implicit influence of quantity on time estimation implies that subjects keep track of magnitude when cued to time, consistently with previous findings reporting that the numerical information interferes with subjects' ability to discriminate duration [4], [5], [12]. Thus, analogical quantity and duration may share information processes resources, behavioral goals and common neural circuits oriented to action, presumably selected through development.

In addition to duration distortions induced by number magnitude, the present findings indicate a strong influence of spatial lateralisation on time judgments: left space biases estimation towards short durations whereas right space biases estimation towards long durations, irrespective on the magnitude of the stimulus timed. This pattern suggests that elapsing time is internally mapped onto spatial representations. We suggest that even the influence of number magnitude on duration judgments could be mediated by spatial attentional factors. In fact, the mere presentation of numbers induces a spatial attentional bias depending on the magnitude of the number: low numbers shifting attention to the left and high numbers to the right space [13]. One could hypothesize that by shifting the focus of attention within a mental representation numbers could indirectly influence time judgments towards underestimation or overestimation. This hypothesis implies a central role of spatial coordinates in the relations linking time with numbers. This issue needs to be investigated more thoroughly at the neural level, to test whether a common foundation for these relationships may be neural processing in posterior parietal cortex, dorsolateral prefrontal cortex and cerebellum, a set of regions involved in cognitively controlled timing [14] as well as in the computation of space and other magnitudes [11].

Overall, these findings fit with the prediction that time could be cognitively represented by means of spatial coordinates, with a left-to-right oriented linear representation, in analogy with numbers and other types of ordered material, such as numbers [15], sound pitches [16], months and letters [17].

In fact, recent evidence has provided direct support to the hypothesis of a left-to-right directionality in time representation. Manipulation of spatial attention by means of optokinetic stimulation biases temporal judgments in healthy subjects according to the side of space where attention is oriented: rightward attentional shifts induce temporal overestimation and leftward attentional shifts induce underestimation of temporal judgments [18]. Moreover, healthy subjects are faster in judging short temporal durations in the left space and long temporal durations in the right space [19].

These evidences challenge traditional views of temporal perception mechanisms, positing that perceptual events are timed by a centralized supramodal clock [6]. The spatial-specific effects of time modulation would be more in accord with the finding that visual events of subsecond duration are timed by visual neural mechanisms with spatially circumscribed receptive fields, localized in real-world, rather than retinal, coordinates [20].

The combination of these findings fit with the proposal that relative time between events may be transformed into specific patterning in neural maps, interpretable with the same mechanisms used to decode cortical representation of spatial images.

## Methods

Twenty-eight right-handed healthy subjects (15 male, age range: 27–39 years), with normal or corrected vision, participated in the experiments after providing written informed consent.

### Experimental procedures

#### Experiment 1

Ten subjects participated in this experiment. They were positioned 60 centimeters from a P791 Dell^a^ computer monitor configured to a refresh rate of 100 Hz.

Visual stimuli constituted by digit pairs (1 and 9, size: 0.8°×0.1°) were presented in the centre of the screen or in the right and left space (5° eccentricity). The duration of the reference stimulus was fixed at 300 ms. The test stimulus was presented after an interval of 1000 ms from the reference one and its duration varied randomly from 150 to 450 ms (excepted the interval of 300 ms) in steps of 20 ms.

Subjects had to indicate the relative duration of the test stimulus compared with the reference one, by pressing either of two response keys using their right hand.

**Figure 2:**
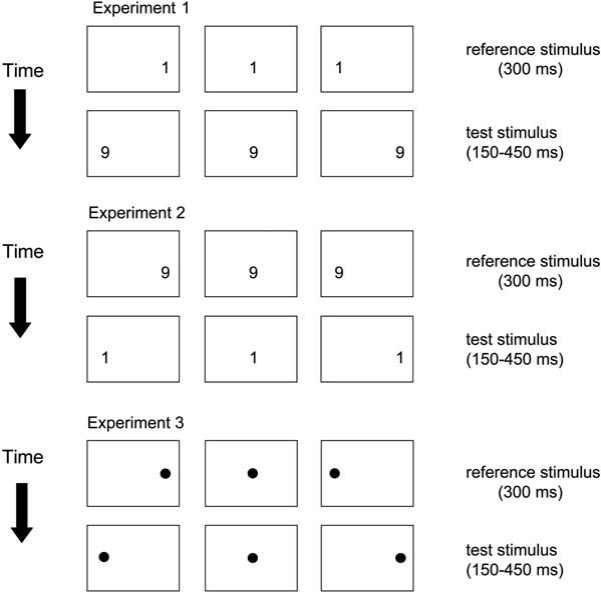
Experimental procedures.

In this experiment, digit 1 was the reference stimulus and digit 9 the test stimulus. Each stimulus pair (reference and test cue) was presented lateralized in the right and left space or in central position in a randomized order. The corresponding conditions were: digit 1 in the left and digit 9 in the right space; digit 1 and digit 9 in central position; digit 1 in the right and digit 9 in the left space (Figure 2).

Each condition consisted of 160 trials of reference and test cues (10 repetitions×16 time intervals).

#### Experiment 2

Ten subjects participated in this experiment. The task and the adopted procedure were identical to that of experiment 1, with the exception that digit 9 was the reference stimulus and digit 1 the test stimulus (Figure 2).

#### Experiment 3

Eight subjects participated in this control experiment, performed to test whether duration judgments are influenced by spatial position of the stimuli irrespective on their numerical magnitude.

Reference and test cues were constituted by identical stimuli (black circles, 1° width).

Time intervals, number of trials and experimental procedure were identical to those of the first two experiments.

Spatial position of reference and test cues was varied, such that they were presented in the left and right space or in central position in an equal number of trials. Therefore, the corresponding conditions were: reference cue in the left and test cue in the right space; reference and test cue in central position; reference cue in the right and test cue in the left space (Figure 2).

Each condition consisted of 160 trials of reference and test cues (10 repetitions×16 time intervals).

### Data analysis

In order to measure the amount of temporal bias observed in each condition, the individual frequencies of “longer” responses were converted into probabilites and a logistic model was fitted to the data of each participant as a function of the duration of the test stimulus (from 150 to 450 ms). This allows to determine the subjective “bisection point” for each condition. This point represents the time interval for which the observer perceives the duration of reference and test stimuli as equal, and is computed as the time interval at which “longer” and “shorter” responses are reported equally often (more specifically when p(“longer” response = .5). A shift in the bisection point in either direction indicates a temporal bias in the corresponding experimental condition.

A paired-t-test was then performed on the individual bisection points to compare the amount of temporal bias in each condition.
